# Exploring the implementation of a new voluntary occupational health and safety program in Ontario, Canada: a thematic analysis

**DOI:** 10.3389/fpubh.2026.1768542

**Published:** 2026-03-03

**Authors:** Kimberly Sharpe, Hannah H. Peck, Suhail Marino, Andrea M. Jones, Christopher B. McLeod

**Affiliations:** School of Population and Public Health, University of British Columbia, Vancouver, BC, Canada

**Keywords:** Canada, injury prevention, knowledge mobilization, occupational health and safety programs, program evaluation, qualitative analysis, safety interventions, workplace

## Abstract

**Objectives:**

The adoption of voluntary occupational health and safety (OHS) interventions has increased over the last three decades. This research explores the practical implications of implementing a new OHS program designed to appeal to a broad range of firms in Ontario, Canada. It also describes the process of providing findings as real-time evidence to program administrators for early program improvement.

**Methods:**

Key informant interviews were conducted with 76 individuals from 71 firms enrolled in the program. Data were thematically analyzed with an emphasis on understanding the challenges and successes participants experienced during their first year in the program, as well as why they enrolled and their overall perceptions of the program. Detailed findings from this study were provided to the program’s administrators during its continued rollout.

**Results:**

The program was well-received and led to early positive changes in OHS. However, there were challenges related to its ‘one-size-fits-all’ nature. Smaller firms with fewer resources, larger firms with more advanced OHS systems and firms with more dynamic working environments faced barriers to participating in the program.

**Discussion:**

OHS programs meant to appeal to a wide variety of firms can be successful but need to be tailored and responsive to differences in firm size, industry and context. Our findings were used to make changes early in the program’s implementation and highlighted the importance of providing timely evidence to improve outcomes and sustainability of OHS programs.

## 1 Introduction

Over the last few decades, the occupational health and safety (OHS) landscape has grown increasingly complex, as workplaces navigate layered regulatory requirements while implementing local OHS initiatives. The OHS agenda has expanded significantly from an initial focus on accident prevention to broader efforts aimed at preventing aiming illness and disease, including addressing psycho-social risk in the workplace (1). Alongside this expanded mandate, the responsibility for protecting workers has increasingly shifted onto employers, who are now expected to self-regulate through internal procedures and safety practices (2, 3). Together, these trends have contributed to a proliferation of OHS interventions, spanning from voluntary activities implemented by individual firms to mandated regulatory requirements (4, 5).

Mandatory interventions are legislated by government bodies and enforced through mechanisms such as audits, inspections, and fines (4). These interventions are typically designed to be broadly applicable across workplaces, including small firms, and are therefore often less sophisticated to facilitate compliance (6). Voluntary interventions may involve OHS improvements implemented by an organization but not part of a broader program or more fulsome programs marketed toward firms aiming to improve their health and safety. These programs operate outside of direct governmental or regulatory mandates and tend to involve more sophisticated requirements. Voluntary programs offered by private agencies frequently target larger firms with advanced safety systems (1). In contrast, voluntary interventions provided by public agencies often aim to accommodate a wider range of firm types by providing simplified or flexible options to cater to varied levels of OHS maturity (7). Voluntary firm-level OHS practices, in particular, are gaining popularity and appear to be driving higher levels of engagement at the local workplace level (8–10).

The ways in which OHS interventions are designed and implemented are increasingly understood to impact their ultimate effectiveness and sustainability (9). In fact, inadequate implementation is often cited as a primary reason why these interventions fail to achieve their intended outcomes (11). Previous research has focused on identifying the contextual factors related to the workplace that affect both the implementation and success of OHS interventions (11, 12). Researchers have highlighted the crucial need for strong organizational commitment to OHS; several studies have identified that strong management commitment and leadership is essential for intervention success, while its absence can be detrimental (7, 13–19). Similarly, worker participation and buy-in are paramount for ensuring that OHS interventions are effectively implemented and sustained (16, 17, 19). Insufficient resourcing, both in terms of specialized personnel and general human and economic resources, can further prohibit effective implementation, particularly among small and medium-sized firms (12, 20). There is limited real-world evidence into how the design of OHS interventions interacts with firm-level implementation contexts (7, 8, 21, 22).

OHS interventions are understood to be complex organizational change processes, where program design interacts with firm-level contextual factors (8, 22, 23). As a result, they rarely follow linear cause-effect pathways as outcomes can depend to the nature of the intervention, the context in which it is implemented, the mechanisms it activates, and the interplay among these factors (21, 24). Investigating intervention design and implementation processes enables deeper interpretation of effectiveness findings, helping to distinguish whether limited impacts are due to the intervention itself or its execution (25–28). At the same time, there have been calls to integrate evaluation evidence earlier in intervention development to ensure they can adapt to and address these contextual differences (25, 29, 30). Formative and process evaluations provide the evidence that policymakers need to make iterative improvements and optimize interventions (21, 22, 30). Furthermore, understanding participant experiences and perceptions can offer particularly valuable insights, especially in voluntary OHS interventions, where context and user experience influences both effectiveness and sustainability (8, 29). Qualitative approaches are well suited to provide insight into the *how* and *why* of intervention implementation and provide support for adapting interventions to real-world settings (31, 32). They are particularly useful for discerning barriers and facilitators to intervention uptake in OHS settings, identifying organizational and contextual factors affecting the intervention, and assessing anticipated sustainability early in an intervention.

This research is part of a five-year mixed-method evaluation of the Health and Safety Excellence (HSE) program, a voluntary OHS program launched in November 2019 by Ontario’s Workplace Safety and Insurance Board (WSIB). This program is designed to accommodate firms of various sizes, industries and contexts across Ontario, Canada. The findings presented in this paper draw on qualitative study examining how the HSE program was implemented in its first 2 years from the perspective of participating firms. Responding to calls for iterative and responsive approaches to evaluating complex OHS interventions, these findings were presented to the WSIB as real-time evidence and used to support early program refinements, improve the experience of participating firms, and inform forthcoming impact analyses in the broader evaluation.

This paper has two aims: (1) investigate the practical implications of designing and implementing a voluntary OHS program meant to appeal to a wide variety of firm sizes, industries and contexts as a one-size-fits-all program, and (2) describe the process of using early evaluation findings to inform program improvement during rollout. This research contributes to our understanding of how the design features and implementation of OHS programs interact with firm-level contextual factors to impact participating firms’ experiences, including differences by size, industry and other organizational characteristics. The findings, as well as the resulting program changes, offer insights applicable to the design and improvement of other voluntary OHS programs and interventions, particularly those that are new or undergoing revision.

## 2 Methods

### 2.1 Study setting: HSE program

In Ontario, the WSIB is responsible for administering wage-loss and medical benefits, and return-to-work support for workers affected by work-related injuries or illnesses. Financed through employer-paid premiums, the WSIB operates a no-fault, collective liability insurance system serving more than 5 million workers across over 300,000 workplaces (33). The WSIB developed and launched the HSE program, a new voluntary OHS program, meant to replace several existing OHS programs. One of the aims of the program is to avoid an overly prescriptive or check-list driven approach, which had been of concern in previous programs.

The HSE program comprises 41 OHS topics (e.g., control of hazards; management review) organized across three levels: foundation, intermediate, advanced (Table 1). The HSE program is designed to support firms to develop their OHS systems as they progress through topics and levels. While the broad structural components build on previous WSIB OHS programs, the HSE model introduces greater customizability by allowing firms to select which topics, and how many, they complete each year as well as flexibility in demonstrating how topic requirements were met.

**Table 1:** List of HSE program topics by topic level.

Level	Topic
Foundation	Leadership and commitment
	Health and safety responsibilities
	Health and safety communication
	Health and safety participation
	Hazard identification^1^
	Hazard reporting^1^
	Workplace inspections^1^
	Risk assessment
	Control of hazards basics^2^
	Control of hazards^2^
	Injury, illness and incident reporting
	Incident investigation and analysis
	First aid
Intermediate	Competency
	Health and safety training
	Legal and other requirements
	Health and safety accountabilities
	Emergency prevention and preparedness
	Emergency response
	Psychological health and safety 1: assessing your risk^3^
	Psychological health and safety 2: reducing your risk^3^
	Return-to-work program requirements, forms and tools
	Return-to-work roles and responsibilities
	Accommodation and return-to-work plans
	Pre-use inspections
	Preventive maintenance
	Control of documents
	Control of records
	Contractor management program
	Workplace health promotion
	Health and safety objectives
	Corrective action
Advanced	Change management and procurement
	Monitoring, measurement and analysis
	Review health and safety trends
	Internal audit
	Management review
	Health and safety continual improvement planning
	External audit
	Networking and peer learning
	Corporate social responsibility

1 At the time of our evaluation, these topics comprised a single topic ‘Recognition of hazards’.

2 At the time of evaluation, these topics comprised a single topic ‘Control of Hazards’.

3 ‘Psychological health and safety 1’ and ‘Psychological health and safety 2’ were added after our interviews had concluded.

To enroll in the HSE program, firms register with a WSIB-approved program provider (Table 2), private health and safety consultants, who guide firms by supporting action plan creation, topic implementation, and evidence submission. Providers offer tiered priced packages, with firms paying their provider directly for their services. Following enrollment, firms complete a self-assessment of their current OHS system intended to identify their appropriate level in the program and develop an action plan, selecting up to five program topics with their program provider. After action plan approval, firms have up to 12 months to complete and validate their selected topics. The HSE program rewards firms for implementing new or significantly improved OHS initiatives; firms cannot use pre-existing initiatives to validate program topics.

**Table 2:** Steps to the HSE program.

Program steps	Implementation and recognition
Step 1: Join	Attend an information session (pre-enrollment) and register with an approved program provider
	Complete safety assessment, select topics and develop action plan with assistance of program provider (5 topics maximum per action plan)
Step 2: Develop	Develop health and safety practices through learning and implementing safety topic(s) with the support of a provider
Step 3: Demonstrate	Demonstrate good health and safety practice by submitting evidence of implementation for review by a program validator
Step 4: Achieve	Achieve recognition by receiving rebates and non-financial recognition (e.g., digital badges)

The validation process aims to ensure that firms’ topics are complete and ‘living and breathing’ in the workplace, meaning they have become a part of day-to-day business activities. Firms submit evidence stories and supporting evidence files through the HSE program online portal. The evidence story is a narrative-based account of how the firm developed and implemented their topic, recounting work performed under each step of the ‘Plan-Do-Check-Act’ model (or other implementation cycle). The WSIB accepts varied forms of supporting evidence files, including videos and photographs. Validators review evidence to ensure topic completion and, where needed, grant firms 60 days to address any outstanding gaps in an additional evidence submission. If the additional evidence still fails to meet the topic requirements, the topic is marked as ‘incomplete’. A workers’ compensation premium rebate is earned for each topic that a firm completes. Completing all topics in a program level is additionally rewarded with a digital badge of recognition.

### 2.2 Research team and evaluation context

This study was conducted as part of an independent evaluation funded through WSIB grants program and was supported by a Project Evaluation Working Group, comprising researchers and WSIB operational staff, and a Project Advisory Committee, which included broader provincial representation, including Ontario Ministry of Labor stakeholders. These groups provided operational and policy context for the evaluation, and were not involved in qualitative data coding, analysis, or theme development. The evaluation consists of three components. The first is qualitative interviews to capture the experiences of participating firms during program rollout and provide program administrators with early feedback. The second is a documentation analysis meant to contextualize program barriers and support qualitative findings. The third is a quantitative analysis of the effectiveness of the program in reducing lost-time workers’ compensation claims.

### 2.3 Recruitment strategy

The analytic team used data on participating firms from the HSE program online portal to develop a purposive recruitment strategy. The goal was to create rich data set representing a diversity of experiences by firm size, sector and region that allowed researchers to discern both recurring thematic patterns and points of divergence (34, 35). Participants from registered firms identified as the most knowledgeable on their firm’s health and safety program were invited to participate in a confidential interview via email. To ensure adequate representation, targeted follow-up emails and phone calls were placed to make final requests for participation and answer any questions.

We conducted interviews with two waves of participants. The first wave was recruited from firms that had enrolled in the HSE program from November 2019 to March 2020; the second wave included firms enrolled from April 2020 to March 2021. For both waves, the target population consisted of firms who had completed at least one program topic. We also recruited a subset of firms who had not completed any topics in wave two in order to better understand the barriers these firms faced. Due to significant disruptions to the Canadian healthcare sector and the designation of healthcare centers as ‘essential services’ during the COVID-19 pandemic, healthcare firms were recruited in the second wave only. Participants from wave one were interviewed between November 2020 to March 2021 and in wave two between October 2021 to December 2021.

### 2.4 Participant and firm characteristics

The final study sample consisted of 76 participants from 71 firms (30 in wave one; 41 in wave two). In five cases, two participants from the same firm requested to participate in a single interview. Of the 76 participants, 39 (51%) had less than 5 years of experience at their current job, while 11 (15%) had 10 or more years of experience (Table 3). In terms of job role, 25 (33%) participants had roles focused specifically on health and safety, while 51 (67%) had roles in administration, human resources, operations, and senior leadership with additional health and safety duties. Of the 25 participants with a primary health and safety role, 2 (8%) were coordinators, 18 (72%) were managers, and 5 (20%) were senior leadership.

**Table 3:** Participant and firm characteristics of the study sample.

Sample characteristics	N	%
Participant characteristics		
Job role		
Administration, office management or coordinators	8	10.5%
Human resources	9	11.8%
Management	9	11.8%
Health and safety^1^	25	32.9%
Health and safety (joint role)^2^	11	14.5%
Senior leadership	13	17.1%
Other	1	1.3%
Years in current role		
Less than 5	39	51.3%
5–9	26	34.2%
10+	11	14.5%
Firm characteristics		
Firm size [full-time equivalent (FTE)]		
Small (1–49 FTE)	23	32.4%
Medium (50–99 FTE)	13	18.3%
Large (100 + FTE)	35	49.3%
Sector		
Construction	21	29.6%
Manufacturing	16	22.5%
Services	13	18.3%
Automotive	4	5.6%
Electrical	3	4.2%
Transportation	4	5.6%
Healthcare	8	11.3%
Municipal	2	2.8%
Program level		
Foundation	6	8.5%
Intermediate	33	46.5%
Advanced	32	45.1%
Current and past involvement in other OHS programs		
None	21	29.6%
1 program	38	53.5%
2–3 programs	11	15.5%
Not reported	1	1.4%

^1^Health and safety analysts, coordinators, managers or directors.^2^Participants with a health and safety role alongside another type of job role (i.e. safety coordination and administrator).

Of the 71 firms, 35 (49%) were large firms with over 100 employees, 13 (18%) were medium firms with between 50 and 99 employees, and 23 (32%) were small firms with between 1 and 49 employees. Construction (21; 30%) was the most represented industry, followed by manufacturing (16; 23%) and services (13; 18.3%). Six (9%) of the 71 firms had enrolled in all foundational level topics, 33 (47%) had at least one intermediate topic enrollment, and 32 (45%) had at least one advanced topic enrollment.

### 2.5 Interview procedure

A structured interview guide was developed in collaboration with the Project Evaluation Working Group and piloted with four firms prior to conducting wave one interviews (Supplementary file 1). Before conducting the second wave of interviews, the guide underwent minor revisions intended to improve clarity and further explore preliminary themes identified from the first wave. The interview guide included questions about firms’ motivation to enroll in the HSE program, OHS changes made, barriers and facilitators to engaging in the program and completing topics, intentions to continue participating in the program, and whether they would recommend the program to other firms. Interviews were conducted by a team member with experience in collecting qualitative data related to workplace health and safety via video conference and lasted 45 min on average. The interview audio was recorded, transcribed verbatim by a professional transcription service and imported into NVivo software, version 12 Pro (QSR International Pty Ltd).

### 2.6 Analysis

We sought to understand challenges and successes participants experienced in their first year of the program, why they enrolled and their overall experience in the program. We began the analysis with structural coding using NVivo software, in which conceptual phrases representing research questions were applied to segments of data (36). This organizational coding facilitated the exploration of commonalities and differences across the research questions guiding the program evaluation and that were of interest to HSE program administrators. The categorization technique provided a preliminary framework for further analysis.

We conducted reflexive thematic analysis, emphasizing iterative and in-depth engagement with the data and researcher reflexivity throughout the analytic process (34, 37, 38). To support rigor, multiple coders familiarized themselves with five interviews from wave one and independently generated preliminary codes through open coding of the same transcripts, documenting reflections and analytic decisions through memos (39). Weekly analytical team meetings were held to generate reflective dialog, resolve interpretive differences and reach shared understanding (38, 40). A coding manual was developed, starting with the preliminary codes, including definitions and examples for each code. For the remaining interviews, team members reviewed each transcript before coding and documented any coding additions or revisions in memos. The coding manual was iteratively refined throughout the analysis as the team deepened their interpretive engagement with the data. The dataset was evaluated on the richness, depth and recurrence of patterns across participants in line with reflective thematic analysis (35, 40).

Following wave one coding, the team examined patterns across and within the dataset to develop an initial set of themes and sub-themes (38). This iterative process involved refining themes and sub-themes, with the team repeatedly reviewing coded segments, full transcripts, candidate themes and emerging thematic groupings. The revised coding manual was applied to the second wave of participants and additional refinements were made following team agreement. As with the first wave, coders again reviewed each transcript before coding and kept detailed reflexive memos. Cross-wave comparison supported the stability of themes as there were no significant differences in themes or codes, and as such, the findings from the two waves are reported collectively. Study implications were developed collaboratively by the full investigative team.

Throughout the evaluation, we engaged the Project Evaluation Working Group to review key components of the study design, such as the interview guide, and to validate the credibility and relevance of emerging findings. The research team also consulted the Project Advisory Committee, which included senior WSIB leadership, the Chief Prevention Officer and Assistant Deputy Minister for Ontario, and other governmental subject-matter experts in OHS program management and delivery. Additionally, we presented our initial findings at the mid-point of our qualitative study. These consultations helped ensure that the evaluation was grounded in operational realities and supported the use of findings to inform program improvements and changes to program design and delivery.

As part of the broader evaluation strategy, we also conducted an in-depth documentation analysis in the third year of the evaluation, the purpose of which was to provide methodological triangulation for our qualitative findings (39, 41) and investigate the effect of specific program changes. Using a systematic approach, this review examined program materials and resources, 300 evidence submission records, validator correspondence, and documentation of program changes (42). The focus of the analysis was to corroborate interview findings and provide an understanding of how participant-reported challenges and experiences were reflected in the evidence submission and validation processes. This documentation review also generated evidence of how interview feedback was shaping tangible program improvements during the early stages of rollout. Together, the subject-matter consultation and documentation triangulation strengthened the credibility and robustness of the findings presented here, and underscored their importance for supporting program improvement.

## 3 Results

The findings are presented in two parts. First, we examine the practical implications of designing an OHS program intended to accommodate all types of workplaces. Second, we explore how participants perceived the program, including whether they intended to continue or recommend the program to other firms. Our findings highlight that firms which did not fit the program’s standard framework, such as smaller, less resourced firms or firms with developed OHS systems, faced difficulties progressing through the program. We describe these challenges both at the program level and at the firm level, illustrating the limitations in designing a ‘one-size-fits-all’ approach (Table 4).

**Table 4:** Themes related to the implementation of the HSE program.

Theme	Sub-theme	Code
Structural guidance and capacity-building		Roadmap toward a full program
		Supporting foundational-level firms
		Desire for robust entry assessment
		Revisiting foundational OHS
Program design and OHS maturity tensions	Continual improvement expectations and value assurance	Focus on ‘new initiatives’ restrictive
		Concerns about aging out
		Importance of maintenance activities
		Return on investment
	Complex organizational structures and program fit	Jurisdiction-specific compliance burdens for national/international firms
		Challenges for nested organizational structures
Balancing flexibility with practical implementation		Guidelines facilitate implementation
		Narrative-based evidence approach
		Preference for clear, concrete examples
		Validator discretion causing inconsistencies
		Adaptability to industry context
Implementation success tied to organizational capacity	Internal expertise	Experienced OHS personnel
		Translating guidelines into practical changes
		Encountering early barriers
	Resource constraints	Wearing ‘multiple hats’/competing demands
		Single pathway challenged small firms
	Essential but inconsistent provider support	Proactive and knowledgeable providers
		Tiered support packages creating uneven access
		Implementation demands diminish cost-effectiveness
Success reinforces participation	Safety improvements and skill-building	Improvements in OHS and safety culture
		Supporting proactive OHS approaches
		Building competence (‘learning to do things well’)
	Recognition and leadership	External recognition
		Championing safety
		Financial incentives reward implementers, motivate leadership

### 3.1 Complexities in designing an OHS program aimed at all types of workplaces

The HSE program appeared to work best for firms at the foundational and intermediate level, who benefited from the program structure and had the internal resources available to invest in the program. Smaller, less resourced firms, firms with well-developed OHS systems and firms with a national or international reach encountered more challenges in making the program fit their workplace contexts.

#### 3.1.1 Program structure

The HSE program’s topic levels from foundational to advanced, emerged as one of its most appreciated features. As one participant noted, the structure conveyed the sense that a full program could be built by advancing through the levels:

And I like the idea that you can continue working on different-- you can continue in different areas where you can improve. So that is-- it’s excellent ‘cause it’s not just one time and then you kind of forget about health and safety. Here you build the whole, you know, from the foundation, the medium and then-- there’s all the different stages in the program, you see how the safety improves (Human resources manager at a large manufacturing firm).

Firms starting their health and safety journey emphasized the benefit of having guidance. Participants at the foundational level, especially those from small and medium sized firms, valued the opportunity to access reliable guidance and resources, including from program providers, as they began building the fundamentals of OHS in their workplaces. This reduced the need to search for and interpret the quality of publicly available OHS and regulatory information. One participant from a large construction firm found that this guidance allowed their firm to be more proactive, rather than reactive, when it came to health and safety:

Because what I find is when you’re first starting out, as much as we had a lot of safety background, this really helped—especially, a small-to-medium-sized business, stay on top of their safety. Because it provided that guidance, right. So without that, you’re kind of—you’re just going based on okay, well, if I get a Ministry inspection they’ll tell me what I’m doing wrong or not. Whereas we are a lot more proactive (Senior leadership at a large construction firm).

While many participants appreciated the ability to tailor participation to their current level, others expressed concern that a lack of OHS expertise, or limited understanding of their firm’s position in its health and safety journey, could lead to missteps. Some participants felt the self-assessment they completed when registering in the program could be more robust. One participant noted that firms without a clear sense of their OHS maturity might inadvertently begin at a more advanced level, potentially missing fundamental elements critical to building a robust system:

I think for this to move forward it would be really good to have companies do a self-audit of where they are with the other stuff and then add to their program. Because it’s like brushing up and fluffing up their program, basically, right. So it’s like for some they’re starting in the middle and not realizing that the basics are not done (Senior leadership at a medium services company).

While the program structure supports stepwise progression, it also allows firms to revisit foundational OHS issues to address ‘inconsistencies’ or add missing ‘building blocks’ in pursuit of a more comprehensive system. This was evident among firms with developed OHS, some of which identified significant gaps and chose relevant foundational or intermediate topics to target specific areas for improvement.

‘You can take everything that you’ve had and start over.’ Those were the words and that’s what kind of captured us and made us think okay, this will be a good thing for our company. We can make sure we’ve got everything in place and we get rewarded for it in the end (Human resources manager at a medium manufacturing firm).

However, while some participants understood and leveraged this feature, others interpreted it more narrowly, as permitting only the initiatives that were entirely ‘new’ to their organization, rather than strengthening existing OHS areas. Making effective use of this flexibility also required participants to have sufficient experience and knowledge to accurately assess their firm’s OHS maturity and identify areas in need of review or significant enhancement.

The program’s emphasis on implementing ‘new’ or significantly improved health and safety initiatives was perceived as restrictive by some firms, especially those from larger and more advanced firms. These participants found that the program was better suited to firms building the foundations or addressing gaps, rather those with mature systems already in place. Furthermore, advanced topics were often viewed as time-consuming to implement, as they required substantial and complex change to already established systems. This created difficulties for some firms in completing all five selected topics within the allotted action plan timeframe, limiting their ability to receive the maximum rebate.

A number of participants from large firms reported enrolling in the HSE program to be recognized as safety leaders in their industry. However, because these firms often had advanced OHS with well-established policies and practices across many topic areas, they felt they would quickly ‘age out’ of what the program could offer. This concern was raised by participants in the electrical sector, who believed that firms in their industry, already subject to stringent standards and regulations, might struggle to meaningfully engage in the program. Participants from firms with developed OHS felt there was ‘no value add-on in going back and redoing everything.’ Instead, as one participant from a large healthcare firm explained, they preferred opportunities to audit or maintain what is already in place in order to support continual improvement and assess the effectiveness of their existing policies and practices:

And there’s no maintenance piece, which you could do five topics this year. Move on next year, do another five and you totally forget about the five you did the year before. There’s no requirement for you to look at those or check to see if they’re working properly or see if you can improve on them. There’s no requirement for that. And so that’s why I don’t call it a system because that system component is really missing from that program (Manager at a large healthcare firm).

In these instances, participants from firms with developed OHS expressed frustration with what they perceived as a large amount of work without commensurate rebates and health and safety improvements. In particular, even with the rebates, some of these participants described their participation as ‘not a good return on investment.’

Similarly, HSE program requirements posed challenges for some participants from nationally or globally operating firms. These firms were interested in topics that could help them understand the Ontario regulatory environment, where the HSE program is offered. However, many already had comprehensive OHS standards and policies designed to meet the most stringent regulations across all jurisdictions in which they operate. In these instances, they were required to create Ontario-specific documentation to satisfy HSE program requirements, even when they believed their existing practices were more rigorous. The burden of maintaining jurisdiction-specific documentation was cited as a barrier to continued participation in the HSE program. One participant described challenges in building and maintaining Ontario-specific OHS documentation that felt misaligned with their broader context as a national firm:

[…] but they may not actually be reflective of what a national company would require. So there was a lot of stuff that would have to be made specifically for an Ontario-based company which I don’t think is beneficial to bigger companies who are national. […] So I’m constantly having to build its own, you know, now that we’re back in this type of a program, we’re constantly having to go back and build in the Ontario legislation. Which is, you know, frankly very annoying and hard to maintain (Health and safety manager at a large services firm).

#### 3.1.2 Topic and evidence submission requirements

The program was designed to be flexible in how firms could meet topic requirements and demonstrate topic implementation. Many participants found the program guidelines valuable in supporting topic implementation, especially those participants with more experience implementing health and safety initiatives or from firms with greater resources. In these cases, participants considered the guidelines a thorough resource that helped ensure they were correctly implementing topics in their firms.

So no, I thought the topic guide was good and it was nice to have the outline and, you know, you could check things off as you’re making your standard to make sure that everything was there (Health and safety coordinator at a large healthcare firm).

In an effort to move away from checklist-driven requirements, the program introduced narrative-based evidence stories, where participants described how they implemented a chosen topic within their firm’s context. These narratives were to be supported by various forms of evidence, such as written documents, videos or photos, to demonstrate how topic requirements were met. However, this flexibility created confusion for some participants, particularly regarding what types of evidence were acceptable, how much was required, and the expected length or tone of the narrative stories. This lack of clarity led to unnecessary stress during the evidence submission process, and, for some, substantial re-work in response to additional evidence requests. Our subsequent documentation analysis corroborated these findings, showing that many firms submitted incomplete or inappropriate evidence that prevented validators from confirming that key topic requirements were met. A few participants also felt that, despite the program’s emphasis on flexibility, the feedback they received from the program validators was ‘nitpicky’, being overly critical or focused on style, for example being told their submission lacked a sufficient ‘narrative story voice’. A common concern was that participants felt they were spending more time preparing submissions than on implementing and evaluating OHS, leading some to view the HSE program as more of a ‘paper exercise’. In some cases, participants submitted copious amounts of documentation in the hope that at least some would meet the criteria, which they saw as an inefficient use of their time. This approach was also observed in the documentation analysis, where some firms responded to requests for additional evidence with large, catch-all submissions.

But there was no, like, there’s no criteria of how long something needs to be. There’s no criteria of what was considered evidence. So you’re basically just shooting in the dark trying to see if, like, this is what they’re looking for. And most of the time it wasn’t. So there’s lack of, like, clear expectation (Health and safety manager at a large services firm).

Participants, particularly those from smaller firms at the intermediate or advanced level, and those with limited in-house health and safety expertise, expressed a desire for more prescriptive guidelines. Some participants noted that policy writing was not their strength and sought clear, step-by-step ‘common-sense’ guidelines in plain language rather than technical jargon. One common challenge among these firms was conceptualizing how they could demonstrate that a topic was ‘living and breathing’ in their workplace. This difficulty was reinforced in the documentation analysis, which showed that the concept of ‘living and breathing’ was described inconsistently by validators. Templates and examples were appreciated, and many participants expressed a desire for more of these practical resources to be made available in future years of the program.

More-- better examples for each section. Like for me I’m visual, so if I see something I would understand it better, other than more words in-- and explanations to bring it all together. So there’s a lot of writing in the program guide and all that. Maybe some examples to make sure you’re on the right track and doing the right stuff (Health and safety coordinator at a small transportation firm).

Similarly, topic and evidence guidelines were often perceived as lacking illustrative examples for smaller firms with fewer resources. For instance, the guidelines referenced roles such as on-site nurses and human resources managers, positions that are rarely present in smaller workplaces. Participants were eager for more materials that reflected the realities of how small firms operate. As one participant from a small manufacturing firm explained:

[…] if I were asked candidly right now if I would refer other small businesses to this program, I would probably say no. And the reason is that there just seems to be this sort of mindset that-- it seems to be designed for companies with much more resources than what I have (Health and safety manager at a small manufacturing firm).

Participants found that the program’s flexibility extended to the validation process, where validators were permitted to use their discretion and consider a firm’s wider context when assessing submissions. While this approach was intended to accommodate diverse workplaces, it sometimes led to inconsistencies. One participant reported submitting identical evidence under two different firm account numbers and receiving approval from one validator, but a request for additional evidence from another. A few participants perceived that validators applied their own individual standards or metrics when assessing evidence:

Because every evaluator had set their own standards and their own metrics. And I may satisfy you, but not the one beside you (Senior leadership at a large construction company).

Furthermore, some participants felt that the program’s evidence requirements and validation process emphasized specific language, phrases or approaches that did not align with their ways of working or with broader industry norms. This led to the conclusion that the HSE program was less adaptable to their industry context. Although participants were supported by program providers, challenges also arose when providers were unfamiliar with the participant’s business or industry. In such cases, providers struggled to offer relevant guidance provide adequate support. For example, one participant in the healthcare sector, whose firm was bound by numerous stakeholders, and felt their provider’s support lacked the necessary nuance, describing it as generic and similar to what is given ‘to the masses’. Similarly, a participant from the retail industry faced difficulties meeting validation requirements across multiple worksites due to limited administrative capacities. With no dedicated ‘office people’ to deliver the program, they had to develop creative solutions to reduce the administrative burden and simplify topic implementation for frontline workers:

But it’s very administrative and, you know, retail is not administrative. If there’s a way we can change this program so it’s not so administrative […] Yeah, we don’t have office people in stores so it’s just we have to be very creative when we send it out to stores and how they can implement making it very simple for them (Health and safety manager at a large services firm).

#### 3.1.3 Internal time and resource commitments for program progression

Having the necessary resources, expertise and experience was critical to successfully implementing the HSE program. Participants from small, medium and large firms consistently emphasized the value of having dedicated health and safety personnel, including professionals or those with prior OHS experience. Across the board, participants found the program workload more demanding than they had expected or planned for. For firms with few or no dedicated OHS staff, this workload was particularly burdensome. Many described struggling to complete topic requirements while managing their regular job responsibilities. This was especially true in smaller, less resourced firms, where employees often wore ‘multiple hats’ and had to balance health and safety tasks with their other duties. A few participants noted that their day-to-day work suffered as a result, or found that program deadlines were difficult to meet due to competing priorities. Those early in their OHS journey also expressed doubts about their ability to maintaining existing OHS efforts while continuing to enroll in new topics and expand their programs.

The timeline can get thrown off because you think you’re going to do this. But then there’s a different safety fire that you have to put out. And we have limited resources. We’re two people. So again, that can be difficult to address the topics (Health and safety manager at a large healthcare firm).

So-- and finding time. Again, small company, everybody wearing multiple hats. And time. Time is always a factor of having enough time to work on it to do it, to roll it out, to-- yeah (Senior leadership at a small transportation firm).

Participants from small firms with limited in-house expertise and OHS experience also found it difficult to translate program guidelines into practical changes in their workplace. One key challenge was in transitioning from informal approaches, such as one-on-one conversations, to formalized policies, practices and documentation. These participants struggled with the formal nature of the program, especially writing policies and evidence stories. A participant from a small construction firm stated this bluntly: ‘I talk. I don’t type.’

Importantly, participants from these smaller, less resourced firms encountered these barriers at an earlier stage in the program compared to larger or more experienced firms. The customizable nature of the HSE program, while designed to offer flexibility, made developing action plans and selecting topics feel overwhelming for those with limited OHS knowledge, with potential to delay progress. One participant described feeling ‘naïve’ while trying to familiarize themselves with the program and create their action plan:

Found it a little bit tough to understand and follow, you know, the portal and going in and all these different bits and parts. And, you know, worked really hard on building the business case for each [topic] and submitting those. And then once we started working more directly with a [provider] representative, things went much better. I’d have to say, like, I had really good support with moving, you know, just-- I could be totally transparent and say I have no idea what I’m doing … at the beginning I just had-- I was just very naïve to the scale of the scope of it (Health and safety manager at a small manufacturing firm).

Building on these concerns, some participants from small firms felt the program’s workload and structure, offering a single pathway and uniform guidelines for all firms, failed to account for differences in firm size and capacity. As a result, it was seen as being designed with well-resourced firms in mind, making it hard for smaller organizations to progress. One participant shared:

I just think that there’s been a bit of-- this idea that one size fits all or that everybody has almost unlimited resources at their disposal. That is far from the truth. Companies like the one I work for, you know, if they’re hoping to engage them and keep them in the program they’re going to have to consider this. Because I reached the point this year where I was almost regretting joining the program (Health and safety manager at a small manufacturing firm).

Purchasing support from a program provider was a core requirement of the program. Participants from various firm sizes and industries credited their program providers for helping them successfully complete their first year. In these cases, providers were proactive and offered a range of resources to support topic implementation. However, most program providers offered tiered support packaged based on firm size, where smaller firms typically qualified for lower-tier support. While some providers offered more comprehensive assistance regardless of firm size, these packages were often cost prohibitive for smaller firms.

Medium and large firms with adequate resources were more likely to have the financial resources to contract out topic implementation and evidence creation to their provider or external safety consultant, or to hire additional staff. In one instance, a participant initially enrolled at the entry-level support tier, but felt they were ‘basically on their own’ and later upgraded their support package after realizing the program’s workload demands. Others opted to expand their internal teams to meet program requirements:

We just hired a new H.R. person who’s going to help with the admin side of a lot of this health and safety stuff. Because we have the knowledge, like, me personally and a few of my colleagues, Joint Health and Safety Committee. But we also need someone with a bit more dedicated time to it (Manager at a large manufacturing firm).

As a consequence, the cost effectiveness of the program was not always evident to participants, particularly given the additional resources required for implementation. One participant noted that a portion of the rebate essentially covered their wages, rather than being re-invested in the company’s OHS program.

### 3.2 Reception of the HSE program

Despite challenges raised here, participants largely recognized the value in the HSE program, with most intending to continue in the program or agreeing that they would recommend it to other firms. Improvements to their firm’s OHS system and safety culture, either expected or realized, were the primary reason participants gave for remain in or recommending the program. One participant from a small manufacturing firm noted that they reported zero claims in the 10-month period after enrolling in the program, where they would typically have three to four claims. Becoming more proactive in OHS, ‘learning how to do things well’, earning external recognition, and leaning into the opportunity to champion safety in their industry were also given as positive motivating factors. Another participant described the impact of the program on support from senior management:

I had lunch today with the three owners and we talked about my focus areas for next year and instead of me suggesting health and safety excellence, they did. They said we want to continue with the program. We see the benefit of it. We want all of our policies and topics to be this robust (HR director at a medium manufacturing firm).

Similarly, financial incentives were a strong motivator for participants, with rebates perceived as valuable rewards for their efforts and as critical incentives to motivate senior leadership in supporting future OHS initiatives.

However, for some participants, continued participation or recommendations to other firms came with a caveat or they signaled their intention to exit the program altogether. For these participants, the workload and amount of resources required were prohibitive and they raised concerns about the cost effectiveness of their continued engagement. This was particularly evident among smaller, less resourced firms and larger, more advanced firms. In one case, a participant from a medium manufacturing company worried they would have to ‘hire a $70,000 per year health and safety manager’ just to ‘receive a $3,000 rebate at year’s end’. An additional concern among participants from larger firms was that the program not well suited for global or national firms or firms with nested organizational structures, such as subsidiaries, because it was difficult to adapt validation requirements to their circumstances. Participants from more advanced firms also discussed ‘aging out’ of the program and moving on to more advanced health and safety programs. Finally, there were some participants who saw value in the program but indicated they would scale back their engagement in future years:

I’m going to attempt just to work on two, let’s say, every three months and see if that give us the opportunity to complete five topics in the whole year. But not being distracted with five topics at the same time because it’s too much to follow up (Health and safety manager at a medium construction firm).

## 4 Discussion

We investigated firm experiences in a newly implemented OHS program in Ontario, Canada. Our findings highlight the complexities and practical challenges of designing a voluntary ‘one-size-fits-all’ OHS program intended to accommodate a diverse range of firm sizes, industries and organizational contexts. Participants recognized the value of a structured framework that also allowed for customization. The programs’ structured progression offered guidance and supported capacity building, especially for small and medium-sized firms, although flexibility in implementation and evidence at times conflicted with practical operational realities. Program fit also differed substantially by firm size and industry. Implementation success was associated with organizational capacity, with smaller firms facing greater challenges due to resource constraints, while larger firms with mature OHS systems reported concerns about aging out and sought recognition for their ongoing system-level efforts. Some national, global, and industry-specific firms described misalignment with program requirements and their operational contexts and structures. Despite these challenges, most participants remained motivated to continue, citing improvements in safety practices, skill-building, and both external and internal recognition as key drivers of sustained engagement.

We found that the program’s structured tiered progression provided important guidance for participating firms, particularly for those small- and medium-sized firms with fewer financial and specialized human resources to engage in OHS independently. This aligns with previous research showing that owners and managers of small firms often lack foundational content knowledge of OHS rules and regulations (43) and value access to technical expertise (12, 44) and guidance on compliance requirements, which are often perceived as complex and difficult to navigate (45, 46). These findings suggest that the structured design of the HSE program may help mitigate some of the resource and knowledge constraints that smaller firms commonly face, positioning structured progression as a meaningful design feature for supporting OHS capacity building in voluntary programs. Previous research indicates that small firms frequently rely on informal, low-resource approaches to occupational health and safety, and that the implementation of more formal interventions is often constrained in the absence of adequate support mechanisms (43, 47). Importantly, our research found that the structured nature of the program assisted firms in transitioning from informal risk management practices to more formal processes.

Our findings illustrate the challenge of striking a balance between flexibility and prescriptiveness in program guidance. Firms in our study experienced challenges interpreting and applying the HSE program’s broad topic and validation guidelines, which were intended to promote customizability and accommodate a wide range of firms. However, flexibility without sufficient guidance created uncertainty about what constituted acceptable implementation or evidence. This challenge is well-recognized in the literature, where overly broad or standardized guidance often fails to account for the practical realities of small firm environments and can limit firms’ ability to translate requirements into effective OHS practices, particularly among those with lower levels of OHS expertise (8, 48, 49).

In contrast, prescriptive or narrowly specified requirements can constrain adaptation, making participation difficult for firms with diverse operational structures or contexts (50). Firms in certain industries, including with more dynamic or decentralized working environments continued to face difficulties implementing their topics and gathering evidence. Workplaces with limited administrative oversight, such retail settings or firms that primarily deploy workers to external sites, struggled to document OHS activities and utilize standardized implementation steps. These challenges are reflected in broader research, finding that dispersed work environments can often inhibit consistent oversight, documentation and implementation of OHS practices (51). This contrasts with industries like manufacturing, where more systematic work processes have historically supported the development and integration of standardized OHS practices (52, 53). Traditional OHS frameworks appear less suited to dynamic, mobile, or multi-site work environments, highlighting the need for participatory approaches in the ongoing design and refinement of interventions to ensure they better reflect the realities of a changing work landscape (9). These challenges resulted in high workloads for firms participating in the HSE program, supporting prior research that has highlighted administrative load as a barrier to implementing OHS and sustaining participation in OHS interventions (44, 54).

Our findings support prior research finding that the implementation of OHS practices in small firms is frequently challenged by limited organizational capacity (43, 44, 49, 55–58). Time and resource constraints, competing demands, and limited internal expertise have previously been identified as major barriers to effective OHS implementation in small firms (45, 55, 56, 59). These constraints led to concerns among participants about sustaining OHS activities while continuing to implement new program topics. Small firms in our study faced challenges early in their participation in the HSE program, including difficulty interpreting program requirements and balancing workload requirements associated with topic implementation within their limited resources. Such challenges are well-documented in previous research, as small firms operate through unique, informal social working relations (43) and can struggle to transition to the formalized processes and extensive documentation requirements embedded in OHS programs (48, 57).

The ability to access additional external resources, such as higher levels of support from program providers or third-party consultants, was limited in smaller firms. The external support of intermediaries has previously been identified as a primary facilitator to participation in OHS interventions for small firms (12, 60) and although small firms have been shown to benefit from the type of personal contact and support offered by intermediaries, they are often less willing or able to pay for such external consultancy services (56, 59, 61). This means that the resource constraints of small firms not only limit their internal capacity for OHS activities but also restrict their ability to supplement that capacity through external assistance (60).

Medium and large firms, particularly those with more mature OHS systems, expressed interest in continual improvement—an important element of effective OHS (62, 63). Consistent with prior research, many of these firms were less focused on introducing entirely new or significantly improved initiatives and more interested in activities such as evaluating, monitoring and maintaining existing systems (8, 50). As a result, the program’s emphasis on ‘new’ or ‘significantly improved’ OHS initiatives created concerns about quickly ‘aging out’ of available topics. In the context of voluntary OHS programs, such challenges with program-firm fit have been shown to undermine long-term program engagement (9, 30). Larger, more advanced firms described motivations aligned with safety leadership and external recognition—drivers found in previous studies of voluntary OHS programs—where reputational and competitive pressures drive participation (5, 7, 45, 46). These findings suggest that improvement-focused interventions may have limited fit for these firms with advanced OHS systems unless they incorporate mechanisms to recognize maintenance and ongoing refinement of existing practices.

Ensuring that the HSE program incorporates maintenance and evaluation elements as ongoing components may help to keep larger, more advanced firms engaged. However, for the most advanced firms, alternate programs may be a better fit. For instance, concurrent with the launch of the HSE program, Ontario’s Ministry of Labor, Immigration, Training and Skills Development introduced the Supporting Ontario’s Safe Employers (SOSE) initiative, designed for firms going ‘above and beyond’ in health and safety with a fully implemented and accredited OHS system. The SOSE initiative may be more appropriate for participants who perceived lack of alignment between the HSE program and their more advanced OHS maturity.

Similarly, large firms with national or global operations encountered difficulties integrating Ontario-specific requirements into their existing systems. Many preferred to adhere to more rigorous international standards, such as ISO 45001, that exceeded the requirements of the HSE program. This reflects what Simpson et al. describe as firms that ‘over-fit’ voluntary management standards. Such organizations often operate under heightened stakeholder expectations to demonstrate continuous improvement, and act as drivers of industry norms rather than passive adopters of existing requirements (8). Recent research has also highlighted the challenges that multinational firms experience in achieving consistent OHS practices across jurisdictions with varying regulatory standards and the utility of using global frameworks to maintain rigor and consistency (64). At the same time, researchers have warned of a potential ‘race to the bottom’ in labor standards (65), where firms exploit jurisdictional differences to benefit from weaker regulations across different areas (66). In this context, Simpson et al. further caution that voluntary programs that do not adequately recognize or challenge more advanced firms may lose their relevance, potentially leading to stagnation OHS performance or disengagement (8). This underscores the importance of ensuring that voluntary programs offer appropriate pathways for those with more advanced OHS systems, such as SOSE in Ontario.

### 4.1 Application of findings

The research team engaged program administrators throughout the project, delivering three rounds of findings to the HSE program leadership in 2021, 2022 and 2023. After the first wave of interviews, we provided preliminary findings through presentations and a report focused on actionable recommendations. Following the second wave, we produced a final qualitative report and presentations highlighting key successes and challenges during the program’s first 2 years. A third report and presentations, specifically linking the documentation analysis with the findings from the qualitative interviews, was provided the following year. These findings were used as real-time evidence to guide iterative improvements to the HSE program.

Informed by our research, the program underwent a significant expansion of supports for participating firms, with small firms recognized as requiring the greatest additional assistance. In response, firms with 1–99 full-time employees became eligible for doubled rebates and a one-time payment of $1,000 upon action plan approval, intended to assist with initial work to implement the HSE program. In addition, a ‘recommended pathway’ of pre-selected topics was developed to facilitate initial engagement for small firms and firms with limited experience in occupational health and safety. A similar stream has been developed for larger, more mature firms that already have Certificate of Recognition (COR®) and ISO 45001:2018. More recently, program administrators, in partnership with a large program provider, have developed a Leadership Stream as an introductory option to better support small firms. This new stream waives registration fees and offers additional supporting during topic selection, a more comprehensive health and safety review, and live webinars for each topic. It offers a simplified entry point and was developed in direct response to challenges and gaps identified through our qualitative findings and documentation analysis. Alternately, for larger, more mature firms the program manual now clearly delineates a pathway to enroll in SOSE as they ‘age out’ of the program.

Given participant concerns about program workload, program administrators divided two of the broader foundational topics into five separate topics to make the requirements more manageable. More detailed instructions on how to meet topic requirements, including illustrative examples, were incorporated into the program instructional manual. These changes were later supported by our documentation analysis, which showed that firms often struggled to demonstrate the full breadth of requirements for this topic in their submissions. To further reduce uncertainty and administrative burden of evidence submissions, the program manual was updated with evidence story templates and illustrative examples for seven topics. Additionally, validators began to conduct clarification calls with firms during the review process. Findings from the documentation analysis suggested that these calls were effective in supporting firms and clarifying requirements.

The submission process itself was streamlined through improvements to the program’s online portal, reducing the number of steps required to upload and submit evidence. In response to inconsistencies in validation outcomes, program administrators introduced a process that ensures a single validator reviews all evidence submissions for multiple business accounts under one employer. The WSIB has also introduced provider rantings to assess the quality and reliability of program providers and address the variability in provider performance identified in our findings.

The responsive use of our research findings during program delivery highlights the value of integrating evaluation into the early stages of OHS program development and implementation. These changes represent the first phase of program evaluation, with future assessments focusing on the impact of the 2022 and 2023 modifications, and, ultimately, the HSE program’s effectiveness in reducing workplace injury and accident rates.

### 4.2 Strengths and limitations

A key strength of the study is its systematic approach to data gathering and analysis. Participants were sampled across a range of job roles, firm sizes, industries, and locations within Ontario, offering a diverse set of perspectives. The use of a structured interview guide allowed for comparison across respondents, especially in areas of interest to the program administrators for program improvement. Structured guides can inadvertently shape responses toward researcher-identified priorities, however we addressed this concern by including probes, follow-up questions, and encouraging participants to provide any additional comments at the end of each interview.

A few limitations should be considered when interpreting our findings. Firstly, the HSE program operates exclusively in the province of Ontario, a single jurisdiction with a provincially administered no-fault employer-funded workers’ compensation system. Our findings may be of particular interest in other jurisdictions with comprehensive disability insurance systems, for instance other Canadian provinces, Australia and Europe, while generalizability may be more limited in jurisdictions with less institutional support. Secondly, our sample largely included firms that had progressed in the program and firms that that did not complete topics were underrepresented; though we attempted to address this by recruiting a subset of such firms in the second wave. Thirdly, we focused on those responsible for program implementation, meaning perspectives from employees, labor representatives and program providers were not captured. Future research should include these stakeholders to better understand buy-in on such OHS programs. Despite these limitations, our qualitative study provides valuable insights into early-stage implementation of a new OHS program, and lays the foundation for ongoing evaluation and program improvement.

## 5 Conclusion

This qualitative study explored practical considerations involved in developing and implementing a voluntary OHS program meant to be ‘one-size-fits-all’ and described how early evaluation findings were used to guide program improvements during implementation. Based on our findings, we offer several recommendations to help support OHS program effectiveness.

1. The first is offering smaller firms additional and targeted support. Voluntary OHS programs may sustain participation more effectively by tailoring internal guidance, external assistance and resources, including financial, to the needs and constraints of small firms. Offering more structured pathways for smaller firms, designed to reflect their specific capacities and constraints, can help mitigate barriers that lead to early attrition.
2. The second is anticipating the needs of larger, mature OHS firms either by incorporating maintenance or evaluation components or by encouraging enrollment in alternative programs to which they are better suited.
3. The third is that evaluators should remain engaged with program administrators throughout the evaluation process to provide evidence-informed updates early in program rollout. In voluntary OHS programs, looking to strike a balance between flexibility and prescriptive clarity, iteratively refining program requirements and guidance can help ensure customizability remains yet account for firms with varying levels of OHS maturity and operating conditions.

## Data Availability

Data collective are qualitative interviews transcripts which are not sharable according to ethical and privacy restrictions. Requests to access the datasets should be directed to chris.mcleod@ubc.ca.
