# Supplementary material for: Exploring the implementation of a new voluntary occupational health and safety program in Ontario, Canada: a thematic analysis
Source: Front Public Health. 2026 Mar 3;14:1768542. doi: 10.3389/fpubh.2026.1768542 (PMC13040360; doi:10.3389/fpubh.2026.1768542)
Supplement: Supplementary file 1 [file Data_Sheet_1.docx]

**WSIB HSEP Evaluation Project - Key Informant Interview Questions**

*The purpose of this interview is for us to understand your perspective as a knowledgeable person regarding your organization’s participation in the Health and Safety Excellence (HSE) Program.*

*For the purposes of the interview, you are interviewee *anonymous ID here*.*

**Questions about the interviewee**

*To start, we have a few questions about yourself, these are help us understand the context of your perspective during this interview*.

1. Can you please describe your title and role at *insert organization name*?
2. How long have you been in this role?
3. What is your educational or professional background?
   1. Do you have any health and safety training or certifications?
4. What are your responsibilities for occupational health and safety?
   1. If they have responsibilities, ask: For how long have you had these responsibilities?
   2. How much of your role focuses on health and safety?
      1. If clarification is needed, say: What proportion of our role is focused on health and safety?
5. How long have you had a role in health and safety?
   1. Within this company? Throughout your career?
6. What are your responsibilities in regards to the HSE program?

**Questions about the organization**

*The next set of questions are to help us better understand your organization.*

1. How large is your organization?
   1. How many people work there?
2. How many locations does your organization have?
3. Does your organization have a unionized workforce?
4. Is or was your organization involved in another health and safety program such as COR, WorkWell, or SafetyGroups?
   1. If yes, ask: For how long were you involved in this program?
5. In your opinion, what is your organization’s motivation for participating in the HSE program?

*Now we have some questions about where your organization is at in the HSE program.*

1. Can you please confirm that after your organization completed the self-assessment tool you were assessed as X level?

*We’ve been provided with a list of topics that your organization has enrolled in and we would like to take a moment to confirm that these are correct before we proceed.*

1. According to our records, you are enrolled in topic XXXX and this topic is currently YYYY (completed, in progress, incomplete*, or deferred)
   1. If they are enrolled in a control of hazards topic, ask: What hazard did you address in the control of hazards topic?
   2. * Note for interviewer - incomplete means: they missed a deadline, or they submitted additional evidence as required but it was not sufficient.
2. In your opinion, why did your organization enroll in these topics?
3. If the answer is too general, ask: Why did your organization choose these particular topics over other ones?
4. Did you find the self-assessment tool helpful in choosing your topics?
5. Were the topics that your organization enrolled in the same as those that were recommended by your program provider?
   1. If no, ask: Why is this?
   2. According to our records, X is your program provider. Is that correct?

**Questions about topics that are complete or in progress**

*The next set of questions are to help us better understand how your organization has responded, invested, or changed its practices or policies due to participation in the HSE program. We will ask the questions about each topic that your organization has completed or nearly completed*.

- *Note to interviewer: Repeat question 14 for each topic that the organization has completed or has in progress (nearly completed).*

1. When rolling out topic XXXX, what do you think was the most important change made by your organization? Why was this the most important change?
2. If clarification is needed, say: Examples of changes include management changes, business changes, procedural changes, or other changes to policy and practice, especially those that impact occupational health and safety.
3. If no significant change was made, ask: Can you tell us why there was no need for a significant change?
4. Thinking of the key change and any other changes you made to implement this topic, can you think of any challenges your organization may have in maintaining/ sustaining this topic?
5. For topics that have not been completed* or have been deferred, can you please tell us more about why this was the case?
   1. For example, did you run out of time to implement topic X? Was any work completed for this topic? Will you be re-enrolling in this topic later on?
   2. * Note for interviewer: topics that have not been completed can mean either expired, deferred, or failed to pass validation even after additional evidence was submitted
6. Did you find the topic requirements clear in the topics guide?
7. Now I’m going to ask you about the types of financial investments that have been or are being made by your organization to roll out the program. These types of investments can be related to specific topics, investments related to the program in general, as well as indirect costs.
   1. Can you please confirm if you spent any money on: (Buckets for them to confirm): Payroll (people or human capital) hours, equipment, infrastructure, training, indirect costs
   2. In terms of time, was the amount of time your organization invested in rolling out the program more, less or about the same as expected?
   3. In terms of money, was the amount your organization invested in rolling out the program more, less or about the same as expected?
   4. Were there any unanticipated costs?

**Questions about barriers and facilitators to ongoing success**

*In this section of the interview, we are trying to understand what helps or hinders organization’s success in the HSE program. We are going to start by asking you about factors within your organization, followed by factors related to working with your program provider, and finally factors related to the WSIB. For each one, we will start by asking about factors that were helpful to your success in the program and then we will ask about factors that made it challenging.*

1. What were the most important factors internally that have helped your organization to progress in the program?
2. If clarification is needed, say: a factor is anything that has helped your organization to progress through the program.
3. What made it difficult internally for your organization to progress in the program?
   1. If only COVID-19 related factors are discussed, use probes to identify non-COVID-19 related challenges.
4. Now thinking of your program provider, what were the most important factors that helped your organization to progress related to your program provider?
   1. What sort of things did your program provider offer to you or do that were helpful?
5. Were there any challenges related to your program provider that made it difficult to progress in the program?
   1. If only COVID-19 related factors are discussed, use probes to identify non-COVID-19 related challenges.
6. Now thinking of the WSIB, what were there the most important factors that helped your organization to progress in the program related to the HSE program resources, tools, or support provided directly by the WSIB?
7. What sort of things did the WSIB offer or do that were helpful?
8. Were there any challenges related to the HSE program resources, tools, or support provided directly by the WSIB that made it difficult to progress in the program?
9. If validation feedback has not been mentioned, ask: Did you receive any validation feedback? (If yes), How helpful did you find the validation feedback?
   1. If validation feedback mentioned, ask: How helpful did you find the validation feedback?
10. If only COVID-19 related factors are discussed, use probes to identify non-COVID-19 related challenges.
11. Were there any other things that were helpful or challenging that we haven’t discussed?
12. Thinking of the barriers and challenges that you have mentioned, what changes could be made to the program to improve your organization’s experience?
13. Can you think of any other changes that could improve the program overall?
    1. If clarification is needed: this can be in terms of what’s required in the program or how you progress in the program.
    2. If topic content is not mentioned, ask: Can you think of any changes that could be made to the topics themselves?
       1. If clarification is needed, say: For example, was any topic too large, or did you think that a topic should be added or removed from the program?

*The next set of questions are to help us better understand how your organization has become safer by rolling out the HSE program.*

1. Overall, how do you think the program has impacted health and safety at your organization?
   1. Is there anything else that the HSE program could do to help your organization be safer?

Note to interviewer: If the interviewee answered ‘Yes’ to question 9 ask question 28. Otherwise skip to question 29.

1. You mentioned that you also participated in *insert other program name*. Do you think that the HSE program has led to changes that would not have happened under *insert other program name*?
   1. Why or why not?

**Closing questions**

1. Can you tell us about your organization’s future plans for improvements to health and safety?
   1. If clarification is needed, say: we’re interested in both what you have planned next for the program but also more generally what you have planned overall in terms of health and safety.
   2. If they do not mention whether they are continuing, ask: Are you continuing with the HSE program?
2. Would you recommend this program to other organizations that are similar to your own? Why or why not?
3. Is there anything else you would like to tell us about your experience with the HSE program?
4. This is the end of the interview. If we have any questions about your answers that we discussed today, would it be ok to contact you again?
5. Do you have any further questions for us?

*Thank you for your time this concludes the interview.*
